# Crop use structures resource selection strategies for African elephants in a human‐dominated landscape

**DOI:** 10.1002/ece3.11574

**Published:** 2024-06-25

**Authors:** Nathan R. Hahn, Jake Wall, Kristen Deninger‐Snyder, Kate Tiedeman, Wilson Sairowua, Marc Goss, Stephan Ndambuki, Ernest Eblate, Noel Mbise, George Wittemyer

**Affiliations:** ^1^ Department of Fish, Wildlife, and Conservation Biology Colorado State University Fort Collins Colorado USA; ^2^ Graduate Degree Program in Ecology Colorado State University Fort Collins Colorado USA; ^3^ Mara Elephant Project Narok Kenya; ^4^ Grumeti Fund Mugumu‐Serengeti Tanzania; ^5^ Max Planck Institute of Animal Behavior Konstanz Germany; ^6^ Wildlife Research and Training Institute Naivasha Kenya; ^7^ Tanzania Wildlife Research Institute Arusha Tanzania; ^8^ Save the Elephants Nairobi Kenya

**Keywords:** African elephant (*Loxodonta africana*), GPS telemetry, human‐wildlife interactions, movement ecology, resource selection, space use, spatial risk

## Abstract

To conserve wide‐ranging species in degraded landscapes, it is essential to understand how the behavior of animals changes in relation to the degree and composition of modification. Evidence suggests that large inter‐individual variation exists in the propensity for use of degraded areas and may be driven by both behavioral and landscape factors. The use of cultivated lands by wildlife is of particular interest, given the importance of reducing human‐wildlife conflicts and understanding how such areas can function as biodiversity buffers. African elephant space use can be highly influenced by human activity and the degree to which individuals crop‐raid. We analyzed GPS data from 56 free‐ranging elephants in the Serengeti‐Mara Ecosystem using resource selection functions (RSFs) to assess how crop use may drive patterns of resource selection and space use within a population. We quantified drivers of similarity in resource selection across individuals using proximity analysis of individual RSF coefficients derived from random forest models. We found wide variation in RSF coefficient values between individuals indicating strongly differentiated resource selection strategies. Proximity assessment indicated the degree of crop use in the dry season, individual repeatability, and time spent in unprotected areas drove similarity in resource selection patterns. Crop selection was also spatially structured in relation to agricultural fragmentation. In areas with low fragmentation, elephants spent less time in crops and selected most strongly for crops further from protected area boundaries, but in areas of high fragmentation, elephants spent twice as much time in crops and selected most strongly for crops closer to the protected area boundary. Our results highlight how individual differences and landscape structure can shape use of agricultural landscapes. We discuss our findings in respect to the conservation challenges of human‐elephant conflict and incorporating behavioral variation into human‐wildlife coexistence efforts.

## Graphical abstract

We assessed patterns of similarity in resource selection across individual elephants linked to crop use, and identified how fragmentation influenced spatial variation in crop selection across the landscape. In areas with low fragmentation, elephants spent less time in crops and selected most strongly for crops further from protected area boundaries, but in areas of high fragmentation, elephants spent twice as much time in crops and selected most strongly for crops closer to the protected area boundary. Our results highlight how individual differences and landscape structure can shape use of agricultural landscapes.

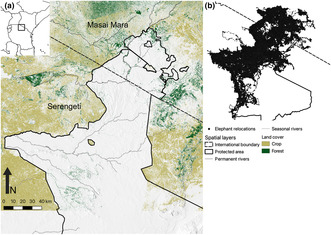

## 1 INTRODUCTION

Animal movement, dispersal, and habitat selection are important determinants of the spatial distribution and dynamics of wildlife populations in heterogenous landscapes (Lima & Zollner, 1996). In agricultural landscapes, behavioral responses are often driven by foraging tradeoffs between accessing highly nutritious crop food and reducing risk from humans (Branco et al., 2019; Simon & Fortin, 2019). The way that animals respond to these tradeoffs can structure ecological processes such as predation, reproduction, and intraspecies competition (Robertson et al., 2013; Sih et al., 2011). To survive in agricultural landscapes animals typically alter their movement behavior as resource availability and risk changes (Broekhuis et al., 2019; Paton et al., 2017), which can affect overall shifts in population distributions (Simon & Fortin, 2019; Veldhuis et al., 2019). The juxtaposition of wildlands and human areas can also influence the relationship between fragmentation and a species' ability to move (Doherty & Driscoll, 2018; Ricketts, 2017). Increasing conflict between wildlife and humans is driving the need to determine wildlife responses to human disturbance (Blackwell et al., 2016; Songhurst et al., 2016). Understanding the impacts of landscape fragmentation on a species' resource requirements and space use is important for designing conservation and management strategies in landscapes where cultivated land is expanding into former wildlands.

Land‐use change by humans has introduced novel resources and altered the risk landscape for wildlife (Gaynor et al., 2019; Simon & Fortin, 2019). For example, crops represent novel, high‐quality food for herbivores but utilization comes at the cost of increased mortality risk (Simon & Fortin, 2019; Sukumar & Gadgil, 1988). Crop use by wildlife is a spatially explicit behavior that is thought to be driven by a risk–reward tradeoff as animals move faster, change to nocturnal activity patterns, and shift space‐use to access crops (Branco et al., 2019; Lewis et al., 2015; Troup et al., 2020). Less understood is the foraging tradeoffs that animals make to seek out crops and the extent to which crop availability affects space use in a population (Pozo et al., 2018).

The ability to collect detailed longitudinal movement data offers powerful insight for the study of spatially structed behaviors (Joly, 2019), such as human‐wildlife interactions (Johnson et al., 2015). Resource‐selection functions (RSFs), which compare landscape characteristics at used sites to those at random sites, is a valuable research tool to study foraging strategies within a population (Leclerc et al., 2016; Sawyer et al., 2006). More recently, interest has grown in using inter‐ and intra‐individual variation in resource selection to understand drivers of population distribution and key areas on the landscape for population persistence (Bastille‐Rousseau & Wittemyer, 2019; Wittemyer et al., 2019). Relating individual heterogeneity in space use to ecological contexts that favor certain tactics or the extent to which individuals can adjust to human pressures can provide further perspective on how tactics may impact population‐level responses to environmental change (Spiegel et al., 2017). Although it can be difficult to discern environmental contexts from remotely collected movement data, spatially explicit behaviors such as crop use are identifiable (Wilkie & Douglas‐Hamilton, 2018).

Crop raiding by elephants is one of the most common types of human‐wildlife conflict in Africa and Asia, and is rapidly increasing as cultivated land expands near protected areas (Shaffer et al., 2019). Elephants typically crop‐raid at night when they are less likely to be detected (Sitati & Walpole, 2005; Tiller et al., 2021; Troup et al., 2020), and in the dry season when crops mature (Branco et al., 2019). The spatial distribution of humans and wildlife can affect the occurrence and intensity of this conflict, but studies have mainly been limited to assessing where conflict events occur on the landscape without information on wider space use surrounding conflict behavior. Conflict hotspots occur in areas with lower densities of human settlements (Denninger‐Snyder et al., 2019), and elephants may also use landscape features such as forest patches and drainages to access crops (Pittiglio et al., 2014; Tiller, 2017). However, conflict hotspots are not necessarily predictive of general elephant space use (Pozo et al., 2018) and elephants have been shown to change foraging strategies to track crop brown‐down (Branco et al., 2019) and shift their ranges closer to crops when raiding (Hahn et al., 2023). Resource selection studies that can link crop use to overall foraging strategies, seasonal factors, and sex would be valuable to understand how conflict is influenced by behavioral and landscape factors, but this approach has been underserved (Mumby & Plotnik, 2018).

Previous research on a subset of Mara‐Serengeti population has shown that the type of protection and management (i.e. strictly protected or mixed use) has strong effects on resource selection, and these effects interact with time of day, season, and sex (Wall et al., 2024). In particular, selection in unprotected areas is strongly differentiated between wet and dry seasons and time of day, suggesting that night‐time crop‐use and human activity may be a key driver in resource selection strategies. Additionally, analyses in this system have demonstrated that the population is influenced by the degree to which individuals crop‐raid, allowing subdivision of these individuals into four behavioral tactics around agricultural use (Hahn et al., 2021). Here we build on this insight to (1) quantify the extent to which using crops impacts overall resource selection strategies and (2) assess how the spatial distribution of crop use relates to the level and structure of fragmentation from crops. Specifically, we test the hypothesis that crop‐use structures selection strategies, resulting in similar resource selection behavior among elephants that use similar amounts of crops each year. Further, we test the hypothesis that selection for crops is influenced by the amount of cultivated land available and level of landscape fragmentation. Specifically, where there are some farms and mosaic of forest patches to facilitate movement, we hypothesize that selection for crops will be greater. We discuss our findings in the context of possible implications on population distributions and conflict risk, management of human‐wildlife conflict across species, and directions for future research.

## 2 METHODS

### 2.1 Study area

**FIGURE 1:**
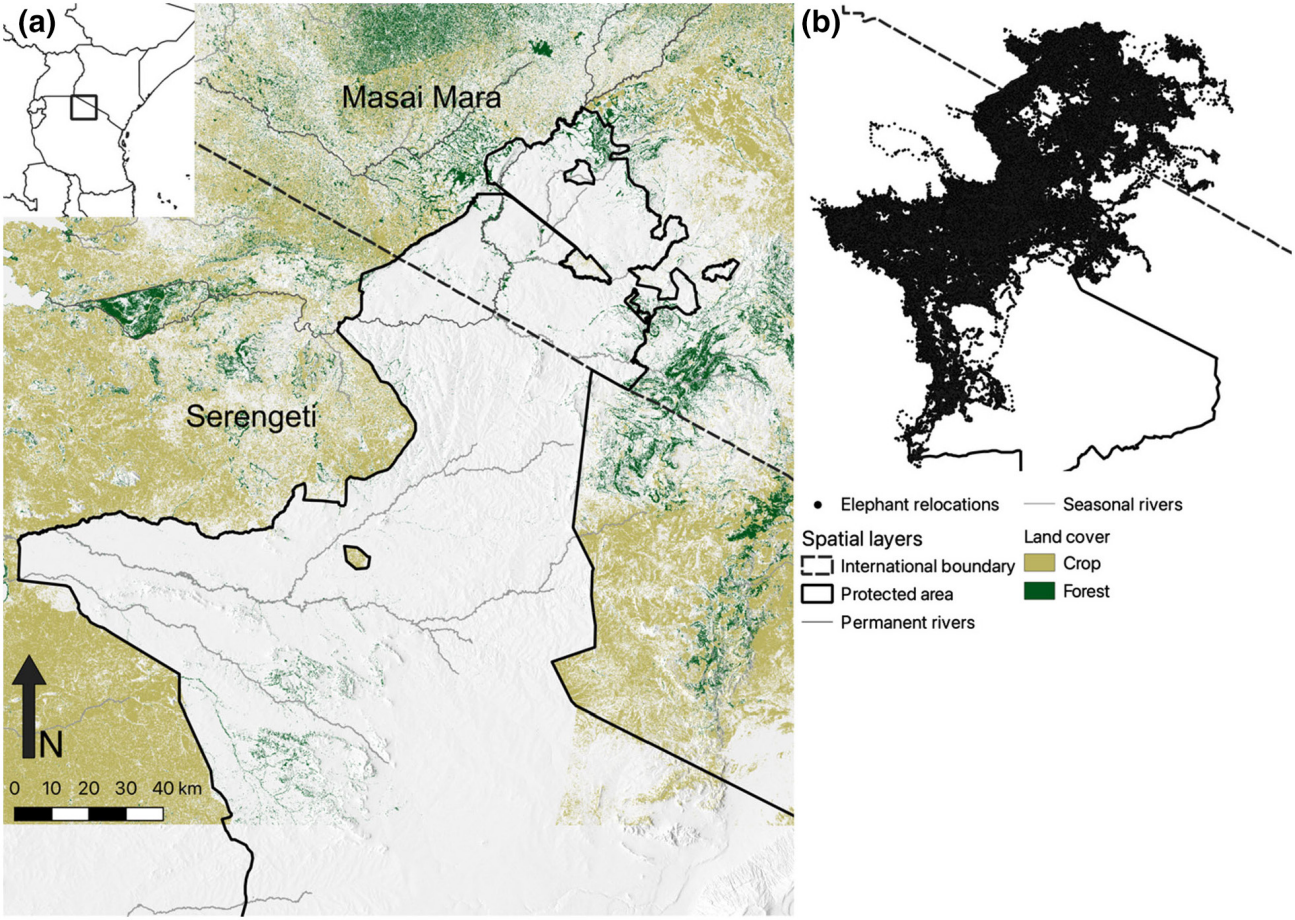
(a) The Greater Serengeti‐Mara Ecosystem showing protected areas, crops, permanent and seasonal rivers, and topography. Protected area boundaries have been simplified. (b) The GPS relocations of the 56 individuals included in the study.

The study took place in the Serengeti‐Mara Ecosystem, a savannah ecosystem in southwestern Kenya and northwestern Tanzania that spans over 40,000 km^2^. Vegetation ranges from large grasslands to woodland, bushland thickets, and afro‐montane forests. The protected areas in the system are made up of the Serengeti National Park in Tanzania and the Masai Mara National Reserve in Kenya, along with private game reserves, community‐managed conservancies and communal lands with managed livestock grazing and no farming (Figure 1). The remaining area is unprotected, comprised of private and community land used mainly for crops and pastoralism. The cultivated land in the region is primarily rain‐fed grain crops and is generally planted in the wet season and becomes mature and is harvested during the dry season. Human‐elephant conflict is highest when crops mature, and incidences of conflict have generally risen over the last two decades (Mukeka et al., 2019; Tiller et al., 2021). Previous work has also found similar levels of crop use between male and female elephants in this system (Hahn et al., 2021; Tiller et al., 2021).

### 2.2 Tracking data

We analyzed GPS data collected from 2011 to 2021 from 57 elephants (204 elephant‐years) that have been tracked as part of long‐term research projects in Kenya and Tanzania (details in Hahn et al., 2021). GPS data collected from females (*n* = 29) represent a family unit while males (*n* = 28) are dispersed and represent a single individual. Locations were filtered to the spatial extent of the study area, subsampled to 1‐h intervals where necessary, and individuals with <95% fix success rates were removed. To assess resource selection across seasons, data on individuals was split into elephant‐year‐seasons (*n* = 401; 201 dry and 200 wet). Due to our focus on crop selection, we also removed tracking data from elephant‐year‐seasons that did not interact with crops (4 dry, 2 wet). After cleaning, the dataset totaled 1,315,403 locations. The region (Mara or Serengeti) for each elephant‐year‐season was determined by the centroid location of their 95% kernel density home range.

### 2.3 Environmental data

Spatial covariates were compiled to analyze resource selection. We created a land cover layer of the Mara‐Serengeti ecosystem using Sentinel‐1 and Sentinel‐2 imagery from 2019 to 2022 at 10 m resolution. Land cover classes were defined by the percent of canopy cover and were categorized as <20% canopy cover (e.g. grassland and savannah) (29.8% of study area), 20%–70% cover (e.g. open woodland and bushland) (46.9%), >70% cover (e.g. forest and bushland thicket) (9.6%), and crops (9.7%). Bare ground, rock, and built land were combined into a single class (1.6%). Land cover classification was performed using random forest classification with 2696 training points and validated using 5‐fold cross validation. Seasons (i.e., wet vs. dry periods) were delineated using NDVI values for the study area using 16‐day MODIS imagery.

The normalized difference vegetation index (NDVI) was extracted from the 250 m moderate‐resolution imaging spectroradiometer (MODIS) vegetation product from 2011 to 2021 at 16‐day intervals and used to determine the coefficient of variation in NDVI for each season‐year. NDVI values were also used to delineate wet and dry seasons using Gaussian mixture clustering (Bastille‐Rousseau et al., 2020). To delineate areas where water was readily available we extracted and buffered rivers and drainages from the global HydroSHED Free Flowing Rivers Network (Grill et al., 2019) by 250 m corresponding to the hourly mean step length for elephants. Slope was calculated based on the 30‐m SRTM digital elevation model (Farr et al., 2007). Settlements were assessed using the Google Open Buildings product for Africa, which classifies permanent structures using machine learning (Sirko et al., 2021). We only included buildings with a confidence level greater than 70% and calculated settlement density using a window size of 250 m corresponding to mean hourly step length of elephants. Roads (Tyrrell et al., 2022) were categorized into primary and secondary roads, and the Euclidean distance to each road type was calculated as a raster with 10 m resolution. The distance to protected area edges was calculated similarly, while allowing for negative values inside the protected areas.

To aid in the interpretation of results, we delineated soft and hard edges between protected and agricultural land in the system based on the crop land cover composition. In the unprotected lands surrounding the Masai Mara, 20.6% of the landscape is converted for crops, compared to 46.9% in the Serengeti landscape. Landscape indices of edge density and contagion index (Hesselbarth et al., 2019; Riitters et al., 1996) of the unprotected land suggest considerably less fragmentation and more interspersion of natural patches in the Mara (edge density = 161, contagion index = 44.5) compared to the Serengeti (edge density = 217, contagion index = 25.4). We considered the Mara region to represent a soft edge between natural and crop land with low fragmentation, and the Serengeti region to represent a hard edge with high fragmentation.

### 2.4 Resource selection functions

To evaluate elephant resource selection relative to crops, we used third‐order resource selection functions (RSFs) based on a use‐available design (Boyce et al., 2002). To characterize availability for each individual‐year‐season, we used GPS locations to define home ranges using 95% minimum convex polygons and generated random locations representing availability within each polygon. The ratio of available to observed points can affect RSF results, so we tested ratios from 20:1 to 50:1 on a subset of individuals and found that model estimates stabilized at 30:1 (Northrup et al., 2013). Available locations were randomly assigned a date and time based on the distribution of the observed locations (Bastille‐Rousseau et al., 2020), and all locations were classified as day (6 am to 6 pm) or night and wet or dry season using dummy coding. All environmental covariates, described above, were extracted for each used and available location. Finally, individual‐year‐season datasets with less than 500 observed relocations or with no observed relocations in crops were removed.

We used generalized linear mixed models with a logit link to evaluate resource selection. Model selection by AICc was performed using a population‐level RSF with a random effect for individual (Bastille‐Rousseau et al., 2020), and the most parsimonious model was then fit to each individual‐year‐season. Coefficient values and 95% confidence intervals were obtained for each individual‐year‐season model. To determine performance of the individual models, k‐fold cross validation using 5 folds was performed for each model.

### 2.5 Drivers of similarity in resource selection

To address our first objective to determine how crop use affects overall resource selection strategies, we applied unsupervised random forest models with 10,000 trees to the RSF coefficients from each individual‐year‐season model. The random forest produces a proximity matrix that represents a measure of similarity between each individual‐year‐season. This proximity measure indicates how frequently a pair of individual‐year‐seasons were classified in the same terminal node of a tree by the random forest algorithm, with values ranging from 0 to 1, where 1 indicates perfect similarity. We then assessed the influence of environmental, seasonal, annual, and individual factors on similarity. We used a beta regression of the proximity metric and weighted the regression by the product of the k‐fold cross validation scores for the associated pair of RSF models (Bastille‐Rousseau & Wittemyer, 2019). We developed five hypotheses based on individual consistency, crop use, protected area use, sex, and season/year that informed our inclusion of each covariate and interaction and fit a single global model to test influence of each factor. The hypotheses and expected effects are shown in Table 1. Covariates included individual (coded as same individual from a different year or different individual), difference in crop use defined as the difference in percentage of observed relocations spent in crops for the associated year‐seasons, region (different region, both Serengeti, both Mara), difference in protected area use defined as the difference in percentage of observed relocations spent in protected, mixed use, and unprotected areas, sex (coded as different sex, both male, or both female), season (different season, both wet, both dry) and year (different year, same year). We also included interactions between individual and season and between crop use and season to assess how proximity changed in relation to season. To help interpret results, we calculated an average absolute effect size for the set of covariates associated with each of the six hypotheses (Table 1).

**TABLE 1:** Hypotheses regarding the drivers of proximity in resource selection strategies among elephants were developed for each set of covariates.

Hypothesis	Covariates	Expected effect
Individual repeatability: Same individual and season (different years) drives proximity	Individual × Season	+
Crops: Similar crop use drives proximity in the dry season	% Ag, % Ag × Season	−
Space use: Similar space use drives proximity	Region + % Protected + % Limited use + % Unprotected	+ Region/ − % Land use
Sex: Same sex drives proximity	Sex	+
Season/Year: Same season or year drives proximity	Season + Year	+

*Note*: Region refers to the Mara and Serengeti regions. Land use was defined as protected and unprotected. All percentages are defined as the difference in percent of relocations between a pair of individual‐year‐seasons.

### 2.6 Spatially explicit crop selection

To investigate our second objective to understand how crop selection varies in relation to protected areas and agricultural composition, we assessed the structure and composition of forest and crops and divided the study system into regions of hard‐edge high fragmentation (Serengeti) and soft‐edge low fragmentation (Masai Mara). Overall, the Mara was characterized by approximately 5 times less crop cover and 2.5 times higher amounts of forest across all buffers. To account for differences in crop structure extending out from the protected area boundary, we stratified the unprotected area of both regions into 1000 m non‐overlapping buffers corresponding to the distance from protected areas. We used 1000 m buffers extending to 3 km, and a final buffer level encompassing elephant range 3–6 km from protected areas. Based on previous conflict assessments, relocations further than 6 km were assumed to be related to dispersal events rather than crop raiding and were discarded (Tiller, 2017).

All elephant relocations were assigned a region and buffer level, which was coded as a multi‐level factor. We fit separate RSFs for each region‐season using generalized linear regressions with a logit link; because not all elephants used all buffer levels, we pooled individuals by region and year. We used crop and buffer level as the two covariates and included an interaction between them to assess how crop selection changed in relation to buffer level. Coefficients and 95% confidence intervals were extracted for each model. To assess goodness‐of‐fit, Area Under Curve values were calculated for each model, and we chose 0.7 as a cutoff value for reasonable model fit (Lemeshow & Hosmer, 1982). Buffers of 250 m corresponding to mean hourly step length produced similar agricultural selection patterns indicating model results were not sensitive to buffer size, but we chose to use 1000 m buffers for clear interpretation.

To help interpret our findings, we calculated landscape metrics within each buffer that could test for differences in the availability of crops and forest (percentage of total land area), and the size and shape of farm and forest patches (mean patch area, mean core area, and perimeter–area ratio) (Wang et al., 2014). We chose to focus on forest due to previous findings that forest patches play a key role as a cover habitat in facilitating crop use (Sitati & Walpole, 2005; Tiller, 2017). All metrics were calculated using the landscapemetrics package in R (Hesselbarth et al., 2019).

## 3 RESULTS

After excluding individual‐year‐season datasets with no observed relocations in crops or with fewer than 500 total relocations in the dataset, 181 elephant‐years from 56 individuals were included in the analyses. There were 176 elephant‐year‐seasons during the dry season and 157 during the wet season.

### 3.1 Individual variation in selection

**FIGURE 2:**
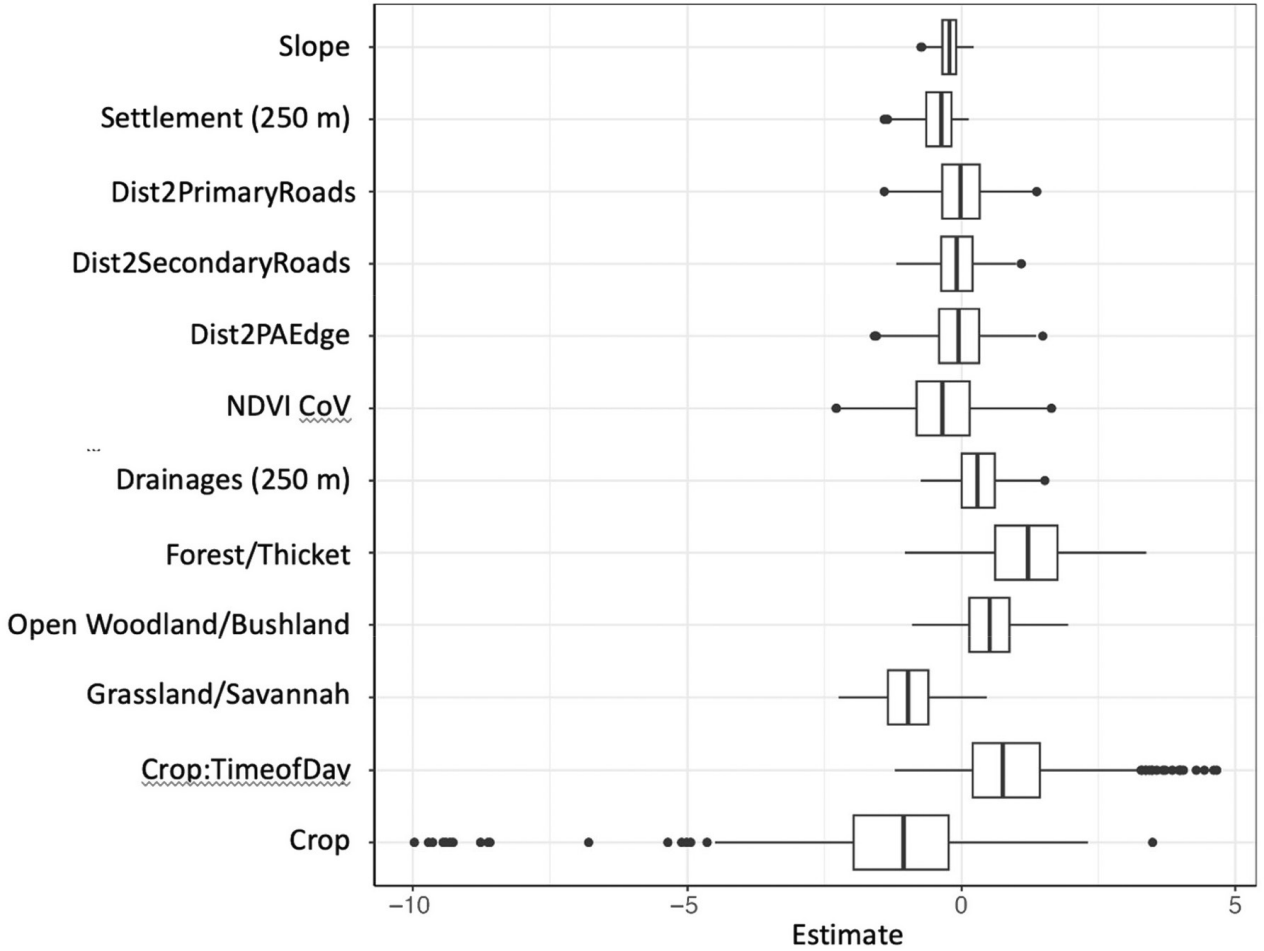
Results of 334 individual‐year‐season RSF models are summarized using boxplots to show the distribution of coefficient estimate for each covariate. Outlier coefficient estimates have been removed to improve clarity of selection trends. Wider boxes indicate greater variability in selection behavior across individuals for that resource. An estimate of 0 indicates no selection, while positive estimates indicate selection and negative estimates indicate avoidance.

We found wide variation in individual selection coefficients. The global model including all covariates and an interaction between crops and time of day was the most parsimonious model and was used to fit individual RSFs. However, while the coefficient confidence intervals for the population‐level RSF did not overlap zero, individual coefficient estimates ranged from negative to positive for most covariates (Figure 2). The greatest individual variation (var = 15.9) was for selection of crops at night (Figure 2). Totally 184 individual‐year‐seasons (55.1%) showed selection for crops at night. It was also notable that when not accounting for time of day, elephants avoided crops on average (Figure 2). The least variation between individuals was selection for slope (var = 0.04) (Figure 2). While the strength of this covariate was comparatively low, all individuals avoided or did not select for slope. Forest (var = 0.31) and drainages (0.28) also showed small variation between individuals.

### 3.2 Drivers of proximity in selection strategies

Proximity analyses indicated similarity between individual elephant‐year‐seasons was weak, with a mean proximity index value of 0.03 (min = 0.005, max = 0.7), demonstrating that the selection strategy of individual elephants varied by year and season. In support of our hypothesis that crop use is a key driver of resource selection strategies, the beta regression model indicated that similarities in overall selection strategies were driven by the individual (mean effect = 0.405), followed by the degree of crop use (mean effect = 0.255). Notably, the similarity for these factors was driven by the dry season (*β*_dry.season:individual_ = 0.772), while effects were greatly reduced in the wet season (*β*_wet.season:individual_ = 0.079). Similar space use (determined by region and time spent in protected, limited use, and protected areas) and sex also had moderate effects on proximity, and in particular time spent in unprotected areas. However, being in the same year or season was the weakest driver of similarity (Table 2). Overall, we found repeatability in an individual's selection strategies between years, but accounting for season showed that this repeatability occurred mainly during the dry season, and there was no similarity in the wet season (Table 2).

**TABLE 2:** Model results showing drivers of proximity between elephants based on resource selection coefficients.

Hypothesis	Term	Estimate	SE	Lwr.95	Upr.95	Mean abs. effect
	(Intercept)	−3.558	0.015	−3.587	−3.529	
Individual repeatability	**subject [same]**	**0.364**	**0.033**	**0.3**	**0.429**	0.405
	**season [dry]:subject [same]**	**0.772**	**0.047**	**0.68**	**0.864**	
	season [wet]:subject [same]	0.079	0.059	−0.036	0.195	
Crop use	pct.crop	−0.008	0.129	−0.261	0.244	0.255
	**season [dry]:pct.crop**	**−0.619**	**0.239**	**−1.087**	**−0.152**	
	season [wet]:pct.crop	0.138	0.193	−0.24	0.516	
Space use	**region [masai mara]**	**0.114**	**0.012**	**0.091**	**0.137**	0.106
	**region [serengeti]**	**0.091**	**0.012**	**0.068**	**0.115**	
	**pct unprotected**	**−0.139**	**0.019**	**−0.176**	**−0.103**	
	**pct protected**	**−0.078**	**0.016**	**−0.12**	**−0.047**	
Sex	**sex [female]**	**0.074**	**0.011**	**0.052**	**0.096**	0.078
	**sex [male]**	**0.082**	**0.011**	**0.06**	**0.104**	
Season & year	**season [dry]**	**0.074**	**0.014**	**0.046**	**0.102**	0.049
	**season [wet]**	**0.063**	**0.014**	**0.036**	**0.091**	
	year [same]	0.012	0.012	−0.011	0.035	

*Note*: Bold lines indicate where 95% confidence levels do not overlap zero. Hypothesis details can be found in Table 1. “subject” refers to the individual elephant IDs. Brackets indicate the factor level. Reference levels for all factors were set as “different”, e.g. “different sex” between a pair of individual‐year‐seasons. Crop use in the dry season was the strongest driver of similarity between different individuals, while within‐individual repeatability between years was strong but only in the dry season.

### 3.3 Spatially explicit crop selection

**FIGURE 3:**
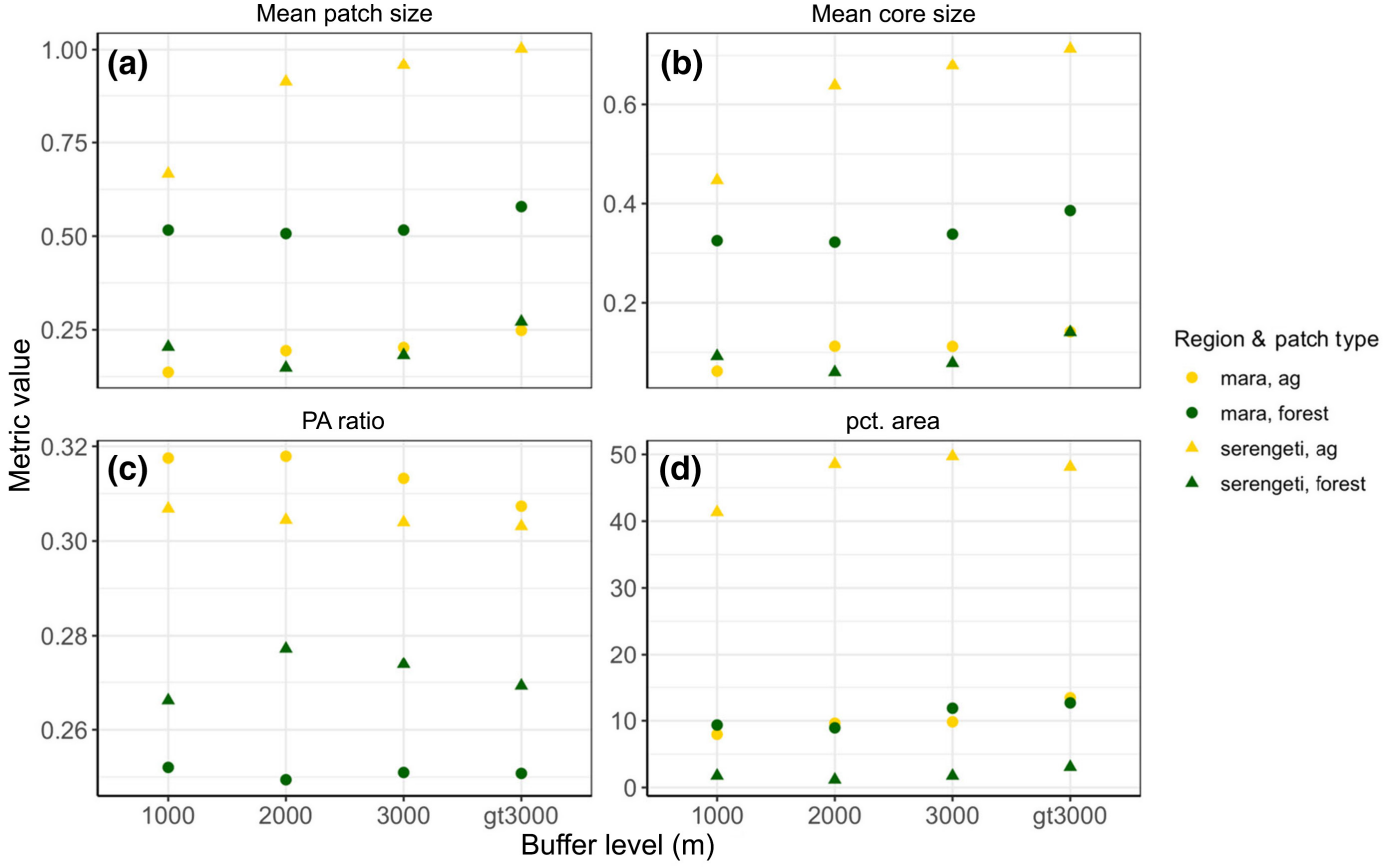
Landscape metrics highlight the strong differences in the wildland‐agricultural interface measured as (a) mean patch size, (b) mean core patch size, (c) perimeter–area ratio, and (d) the percent area covered by crops and forest. Values are shown for each distance buffer level outside the Mara and Serengeti protected areas. Colors correspond to patch type (yellow = crops and green = forest) and shape corresponds to region (circles = Masai Mara and triangles = Serengeti).

Our evaluation of landscape metrics across each buffer showed clear differences in both the extent and structure of crops and forest between the Mara and Serengeti. The first three buffers in the Mara all contained similar amounts of crops ranging from 9% to 11% before increasing in the >3000 m buffer, while the Serengeti saw a sharp rise of 40% crop to 50% crop between the 1000 and 2000 m buffers before leveling off (Figure 3d). Compared to the Serengeti, crop patch area in the Mara were approximately 3.5 times smaller (Figure 3a,b) and slightly more complex (Figure 3c), while forest patches were 2.5 times larger and slightly less complex.

**FIGURE 4:**
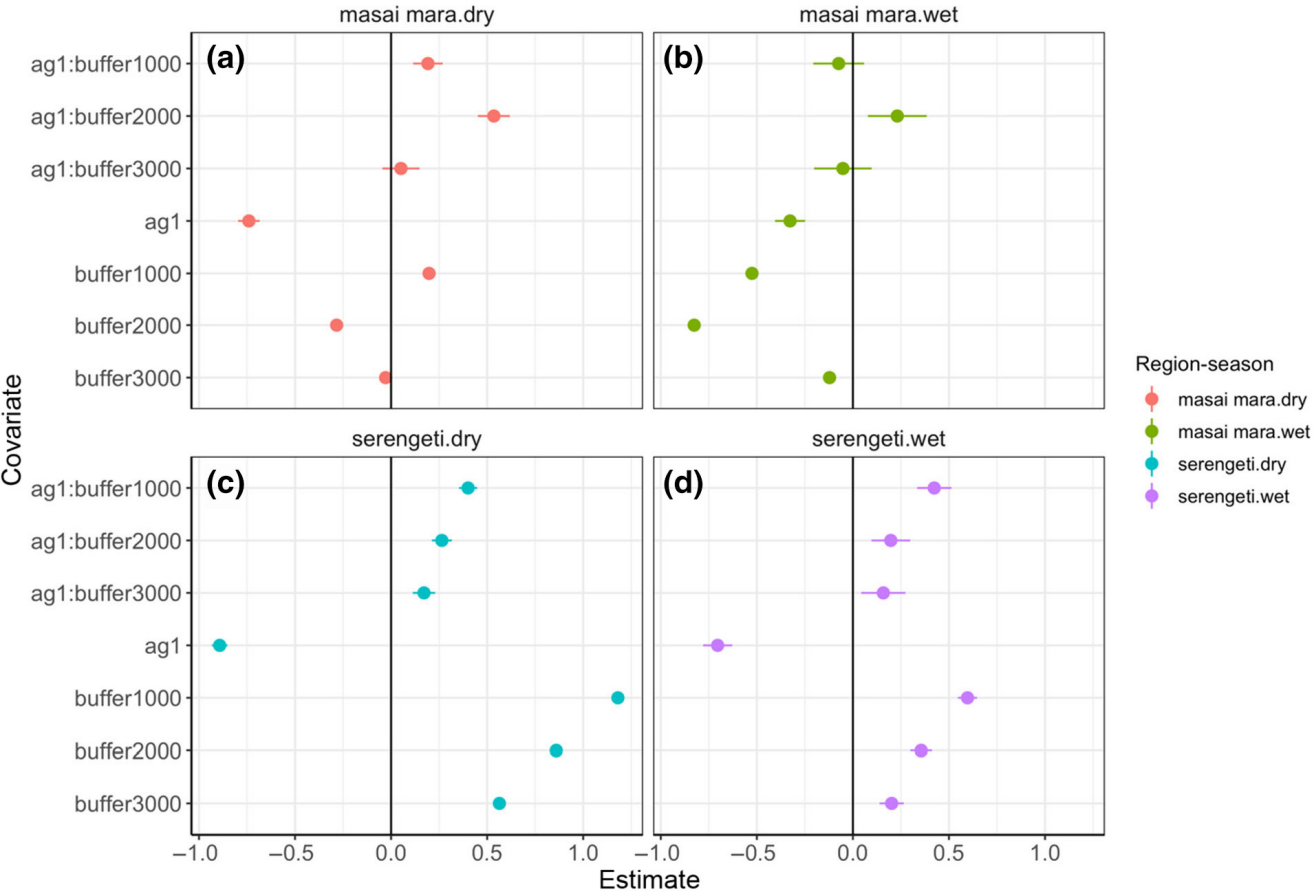
Selection of crops in relation to buffer levels outside protected areas are shown for each region *season (a‐d). Buffers of <1000, 1000 to <2000, 2000 to <3000, and >3000 m from protected area edge were used, with the >3000 m buffer used as the reference level. Bars represent 95% confidence intervals. Selection for crops without considering proximity to protected area edge shows strong avoidance overall. However, when accounting for spatial context, the mara region shows a peak in selection for crops in the 2000–3000 m buffer, whereas selection in the Serengeti peaks at 0–1000 m and tapers linearly.

In our assessment of crop selection related to the landscape structure of crops and forest outside protected areas, we detected different patterns of selection between the soft‐edge region of the Mara and the hard‐edge region of the Serengeti. Here we focus on the dry season when the majority of crop use takes place, but wet season model results are also presented (Figure 4, Table 3). In the Mara, selection for crops during the dry season was strongest in the 2000 m buffer (*β* = 0.535; 95% CI [0.451, 0.619]) followed by in the 1000 m buffer (*β* = 0.198; 95% CI [0.182, 0.213]), but there was no selection for crops in the 3000 m buffer (*β* = 0.051; 95% CI [−0.045, 0.148]) (Figure 4a). This pattern of selection for crops appeared to be the inverse of selection for the overall buffer area (Figure 4b), with the 2000 m buffer being avoided most strongly (*β* = −0.283, 95% CI [−0.303, −0.264]), despite experiencing the strongest selection for crops. In contrast, elephants in the Serengeti during the dry season selected most strongly for crops in the 1000 m buffer (*β* = 0.597; 95% CI [0.547, 0.646]), and this diminished linearly for the 2000 m (*β* = 0.355, 95% CI [0.299, 0.412]) and 3000 m buffers (*β* = 0.202, 95% CI [0.139, 0.265]) (Figure 4c). The selection for the buffered area followed the same pattern, with the 1000 m buffer being selected most strongly (*β* = 1.181, 95% CI [1.157, 1.204]) and diminishing linearly. Notably, in both regions, selection for crops across the entire unprotected area was negative in the dry season. Dry season models (Mara AUC = 0.72, Serengeti AUC = 0.76) had reasonable goodness‐of‐fit, but this was not the case for wet season models (Mara AUC = 0.59, Serengeti AUC = 0.61). Coefficient estimates and 95% confidence intervals for all models can be found in Table 3.

**TABLE 3:** Coefficient estimates are shown for the spatially explicit crop selection model.

Season	Region	Term	Estimate	SE	Lwr.95	Upr.95	AUC
Dry	Masai Mara	ag	−0.74	0.029	−0.796	−0.684	0.72
		buffer[1000]	0.198	0.008	0.182	0.213	
		buffer[2000]	−0.283	0.01	−0.303	−0.264	
		buffer[3000]	−0.028	0.01	−0.048	−0.008	
		ag:buffer[1000]	0.192	0.039	0.115	0.269	
		ag:buffer[2000]	0.535	0.043	0.451	0.619	
		ag:buffer[3000]	0.051	0.049	−0.045	0.148	
	Serengeti	ag	−0.892	0.021	−0.932	−0.852	0.76
		buffer[1000]	1.181	0.012	1.157	1.204	
		buffer[2000]	0.86	0.013	0.833	0.886	
		buffer[3000]	0.564	0.015	0.534	0.593	
		ag:buffer[1000]	0.401	0.024	0.354	0.448	
		ag:buffer[2000]	0.264	0.026	0.212	0.316	
		ag:buffer[3000]	0.171	0.03	0.113	0.23	
Wet	Masai Mara	ag	−0.327	0.04	−0.405	−0.249	0.59
		buffer[1000]	−0.525	0.013	−0.552	−0.499	
		buffer[2000]	−0.826	0.017	−0.86	−0.793	
		buffer[3000]	−0.122	0.016	−0.152	−0.091	
		ag:buffer[1000]	−0.074	0.068	−0.207	0.058	
		ag:buffer[2000]	0.231	0.078	0.078	0.384	
		ag:buffer[3000]	−0.052	0.077	−0.202	0.098	
	Serengeti	ag	−0.704	0.039	−0.78	−0.628	0.61
		buffer[1000]	0.597	0.025	0.547	0.646	
		buffer[2000]	0.355	0.029	0.299	0.412	
		buffer[3000]	0.202	0.032	0.139	0.265	
		ag:buffer[1000]	0.423	0.046	0.334	0.513	
		ag:buffer[2000]	0.197	0.051	0.097	0.298	
		ag:buffer[3000]	0.158	0.059	0.043	0.274	

*Note*: “ag” indicates the crop covariate and “buffer” indicates the buffer level. Brackets indicate the buffer level (e.g. 1000 = less than 1000 m from protected areas). Area under the curve (AUC) values are reported for each of the four models as a measure for goodness‐of‐fit.

## 4 DISCUSSION

Understanding how behavioral and landscape shape the use of cultivated lands by wildlife is of increasing interest, given the importance of reducing human–wildlife conflicts and understanding how such areas can function as biodiversity buffers. Using GPS tracking data from African elephants across two regions with markedly different wildland–agricultural interfaces, we found crop use was a key factor structuring the highly variable resource selection strategies in the study system. Furthermore, the interface between crop lands and natural habitats interacted with proximity to protected area to structure crop use. Our results demonstrate the role individual strategies and landscape structure have on elephant crop raiding behavior, providing information that can help target management interventions.

We found high individual variation in resource selection but identified several covariates to which individuals responded consistently. Across individuals, elephants avoided slope to a similar degree. In addition, drainages and forest cover also showed similar positive selection. In previous research from the Mara region assessing population‐wide trends in selection across land management types, these covariates were also identified as showing little differentiation (Wall et al., 2024). Together, the small variation in selection suggests that slope, drainages, and forest are important drivers for elephant space use across the Serengeti‐Mara ecosystem and should be considered in protecting elephant habitat and building connectivity.

Proximity analyses were able to further assess drivers of similarity in selection by elephants. Intra‐individual similarity between years was stronger in the dry season, while elephants appeared to have different selection strategies in the wet season from year to year. This was in contrast to findings from an elephant population in arid northern Kenya, where dry season resource limitations appeared to drive differentiation in selection strategies and wet season resource abundance allowed more convergence in strategies (Bastille‐Rousseau & Wittemyer, 2019). Notable differences in this northern system includes the much more arid climate and general lack of cultivate. In the Serengeti‐Mara, crops had the highest variation in selection between individuals, similar crop use between individuals in the dry season was a strong driver in similarity in overall resource selection strategies, a factor not structuring dry season resource selection in Northern Kenya. The dry season is when crop use by elephants is highest in the Serengeti‐Mara system and the differences in crop use between individuals are most stark (i.e., those that raid crops and those that do not; Hahn et al., 2021). Wall et al. (2024) found that population‐wide crop selection in the Masai Mara varied most between wet and dry seasons, and our results provide further insight suggesting that crops play an important role in shaping seasonal resource selection strategies in the system. Further exploration is needed on the influence of seasonal dynamics on resource selection and space use, including quantifying and accounting for seasonal conditions when assessing the degree of differentiation observed in strategies.

Previous findings have shown that elephants change their behavior to use crops, including shifting their ranges as crops mature (Branco et al., 2019), changing daily activity budgets (Hahn et al., 2021), and altering their movement patterns prior to raiding (Troup et al., 2020). Our results suggest these behaviors require elephants to adjust their overall foraging strategy during periods of crop use in a relatively similar way. Given the major inter‐individual differences in elephant movement behavior (Bastille‐Rousseau & Wittemyer, 2019), the convergence on similar strategies for utilizing crops indicates opportunities for management‐based interventions targeting a large swath of crop raiding individuals. Given the timing and resource selection behavior of crop raiders are relatively predictable, targeted intervention such as the development of barriers in high‐risk areas or shifting to alternative non‐attractant crops (though see discussions below) could be feasibly implemented in crop raiding hotspots.

We also found strong spatial structuring in crop selection based on distance from protected areas, the extent of fragmentation, and the patch structure of crops and forest. Notably, elephants strongly avoided crops in general, but when looking at selection in agricultural buffers we found that elephants selected and avoided for crops in relation to the landscape pattern of crops in the area. Specifically, with more fragmentation and smaller forest patches (as found in the Serengeti), elephants selected strongly for crops close to the protected area boundary. In the Mara, with larger forest patches and lower density of cultivated land, we found a strong signal for selection in the 2000–3000 m buffer around protected areas compared to other buffers. This could be due to the mean patch and core size of crops, which is low in the 1000 m buffer of the Mara and increased in the 2000 and 3000 m buffer. This suggests that a softer edge between natural and cultivated land can allow elephants to move further into unprotected areas and in turn shift the spatial distribution of conflict risk to farms further from protected areas. The importance of forest patches to this movement behavior also aligns with previous work showing that elephants and other species rely on extant forest to access crops and limit detection from humans (Hahn et al., 2023; Tiller, 2017).

It should be noted that overall crop use per individual was twice as low in the Mara (mean of 2.03% of fixes in crops) than in the Serengeti (mean of 4.76% of fixes in crops), indicating that a soft edge and less overall crops could help to dissuade overall crop raiding. Our results suggest that the landscape structure and the ability of animals to move through it plays an important role in determining where conflict may occur and points to how conflict risk may change as land around protected areas are converted or as corridors and stepping‐stone habitat is established. Overall, conflict management strategies should consider how the makeup of forest and crops can affect the intensity and spatial occurrence of conflict and thereby the types of mitigations, and must be adaptable as landscapes continue to change. For example, in a soft edge when crops and forest are interspersed and conflict is more diffuse, shifting to non‐palatable crops and farm‐based mitigations may be most appropriate, while permanent barriers may be more effective for areas with high amounts of crops adjacent to the protected area.

Several caveats to our approach should be noted. First, while we detected multiple factors that drove similarity between individuals, proximity between individuals was low overall. The proximity values we found were similar to those found in a study on African elephants in Northern Kenya (Bastille‐Rousseau & Wittemyer, 2019), which further supports that elephants generally have wide variation in selection strategies. This variation in behavior will limit the effects of any targeted management interventions. Second, our ability to compare spatially structured crop selection in the Mara and Serengeti is only correlative due to the observational nature of this study. Third, while the low predictive power of the agricultural buffer models was typical for elephants with high individual variation, it limits the extension of these findings beyond the study system. Based on our results, a controlled study or one using simulated landscapes (e.g. Signer et al., 2017) may be valuable to further explore how variation in animal response to fragmentation, forest cover, and protected areas can impact population distributions. This could have applications in limiting human‐wildlife conflict through landscape planning exercises such as wildlife dispersal areas and corridors (Buchholtz et al., 2020).

As cultivated lands are expected to expand and intensity in many parts of the world, information on how species may adapt their foraging strategies and space use in relation to crops will be critical to predict the impacts on populations and manage species persistence in mixed‐use landscapes. While our study focused on elephant crop use, our approach is translatable to other species that are adversely affected by human agricultural and urban development. This may be most productive for species where variation in conflict risk between individuals has already been documented and conflict is concentrated spatially on the landscape, such as carnivore depredation of livestock (Linnell et al., 2008), bears accessing human food resources (Lewis et al., 2015), and herbivore use of crops (Simon & Fortin, 2019). Such investigations can play an important role in informing mitigation efforts and land use planning initiatives that incorporate behavioral complexity in human–wildlife conflict hotspots.

## AUTHOR CONTRIBUTIONS

**Nathan R. Hahn:** Conceptualization (equal); formal analysis (lead); methodology (lead); writing – original draft (lead). **Jake Wall:** Conceptualization (equal); formal analysis (supporting); methodology (supporting); writing – review and editing (equal). **Kristen Deninger‐Snyder:** Conceptualization (equal); formal analysis (supporting); methodology (supporting); writing – review and editing (equal). **Kate Tiedeman:** Formal analysis (supporting); methodology (supporting). **Wilson Sairowua:** Data curation (equal); resources (equal); writing – review and editing (equal). **Marc Goss:** Conceptualization (equal); resources (equal); writing – review and editing (equal). **Stephan Ndambuki:** Writing – review and editing (equal). **Ernest Eblate:** Writing – review and editing (equal). **Noel Mbise:** Resources (equal); writing – review and editing (equal). **George Wittemyer:** Conceptualization (equal); formal analysis (supporting); methodology (supporting); supervision (lead); writing – review and editing (supporting).

## FUNDING INFORMATION

Nathan Hahn was supported by the National Science Foundation Graduate Research Fellowship Program.

## CONFLICT OF INTEREST STATEMENT

The authors declare no conflicts of interest.

## Supporting information


Data S1

## Data Availability

Elephant GPS data have not been archived due to their highly sensitive nature. Interested readers can contact the corresponding author directly for inquiries. Example code is presented in the Data S1.
