# Supplementary material for: Crop use structures resource selection strategies for African elephants in a human‐dominated landscape
Source: Ecol Evol. 2024 Jun 25;14(6):e11574. doi: 10.1002/ece3.11574 (PMC11196896; doi:10.1002/ece3.11574)
Supplement: Supplementary file 1 — Data S1 [file ECE3-14-e11574-s001.zip › SM1_LandcoverLayer_Hahn.docx]

**SM 1: Land Cover Classification Details**

The land cover classification was created using Sentinel-1 and Sentinel-2 satellite data with a Random Forest model on the Google Earth Engine platform.

*Data collection*

We collected 2,574 ground truth land cover data points from 2019-2021, using the following classifications: (1) rock/bare/built, (2) agriculture, (3) greater than 70% cover (forest, thicket), (4) 20-70% cover (shrubland, open thicket, woodland), and (5) less than 20% cover (wooded grassland and grassland). To provide spatial coverage in areas that were difficult to access we scored Sentinel-2 imagery (122 data points) in Google Earth Engine from 2020 and 2021.

*Satellite imagery*

We created a multi-year composite using Sentinel-1 and Sentinel-2 surface reflectance (SR) imagery for 2019-2021. Collectively, the European Space Agency (ESA) twin satellites, Sentinel-2A and Sentinel-2B, referred to as Sentinel-2, have a five day revisit period and 13 spectral bands from visible to shortwave infrared at 10-20 m resolution. Sentinel-2 SR has been processed with an atmospheric correction applied to Top-Of-Atmosphere (TOA) Level-1C orthoimage products. We used a cloud score of less than 30% per pixel to create a cloud mask. We then used the following bands from Sentinel-2: blue, green, red, red edge 1, red edge 2, red edge 3, red edge 4, near-infrared, short wave infrared 1, and shortwave infrared 2. We used VV and VH from Sentinel-1 ascending orbit.

Using these bands, we created the following indices: Enhanced Vegetation Index (EVI), green brown vegetation index (GBVI), Green chlorophyll vegetation index (GCVI), Green red vegetation index (GRVI), Normalized Difference Vegetation Index (NDVI), and Normalized Difference Moisture Index (NDMI). For each band and index we used the 10th percentile, 25th percentile, 50th percentile, 75th percentile, 90th percentile, the difference between the 90th and 10th percentiles, and the difference between the 75th and 25th percentiles for the study period. We also included slope, elevation, and aspect, from the Shuttle Radar Topography Mission (Farr et al. 2007) at a resolution of 1 arc-second (approximately 30m).

*Results*

**SM Table 1**. Accuracy metrics from 5-fold cross validation of the land cover classification. The balanced accuracy for all classes were above 83%.

| **Metric** | **Bare/Built** | **Crop** | **<20% Cover** | **20-70% Cover** | **>70% Cover** |
| --- | --- | --- | --- | --- | --- |
| Sensitivity | 0.7532 | 0.7115 | 0.8498 | 0.8141 | 0.79795 |
| Specificity | 0.9931 | 0.9650 | 0.8175 | 0.9542 | 0.98058 |
| Pos Pred Value | 0.9461 | 0.8465 | 0.7243 | 0.7870 | 0.7870 |
| Neg Pred Value | 0.9617 | 0.9249 | 0.9061 | 0.9611 | 0.9611 |
| Prevalence | 0.1380 | 0.2135 | 0.3607 | 0.1720 | 0.1720 |
| Detection Rate | 0.1039 | 0.1519 | 0.3065 | 0.1401 | 0.1401 |
| Detection Prevalence | 0.1099 | 0.1794 | 0.4232 | 0.1780 | 0.1780 |
| Balanced Accuracy | 0.8732 | 0.8382 | 0.8336 | 0.8842 | 0.8842 |
